# SEQUENTIAL IMPLEMENTATION AS A LEARNING HEALTH ORGANIZATION: THE EQUIPPED GERIATRIC MEDICATION SAFETY MODEL

**DOI:** 10.1093/geroni/igz038.3434

**Published:** 2019-11-08

**Authors:** Ann E Vandenberg, Michelle Kegler, Susan Hastings, Ula Hwang, Camille Vaughan

**Affiliations:** 1 Division of General Medicine and Geriatrics, Emory University School of Medicine, Atlanta, Georgia, United States; 2 Rollins School of Public Health, Emory University, Atlanta, Georgia, United States; 3 Department of Medicine, Duke University School of Medicine, Durham, North Carolina, United States; 4 Departments of Emergency Medicine and Geriatrics, Icahn School of Medicine at Mount Sinai, New York, New York, United States

## Abstract

A learning health organization (LHO) is one that systematically integrates internal data and experience with external evidence to improve internal healthcare practice. Yet collaborative research networks implementing evidence-based interventions across sites with the goal of widespread dissemination are also effectively LHOs. The EQUIPPED (Enhancing Quality of Prescribing Practices for Older Adults Discharged from the Emergency Department) network formed to address an important public health issue: potentially inappropriate medications (PIMs) prescribed to older adults at discharge from hospital Emergency Departments (ED). EDs nationwide serve increasing numbers of older adults but lack clinical decision support to avoid prescribing PIMs associated with adverse events including hospitalization and death. The EQUIPPED geriatric safety program was adapted from the VA and implemented sequentially at three different academic institutions sharing the same electronic health record (Epic)(AHRQ R18HS24499). Implementation challenges, solutions, and innovations informed successive iterations. Using the Replicating Effective Programs framework, we conducted a process evaluation using data from implementation team focus groups (n=3), meeting minutes (n=98 hours), and organizational profiles (n =3) to understand how organizations working together within a research network build an intervention package for program scale-up. We present structural characteristics of the three organizations, implementation steps as they developed across three sites, and the resulting process protocol and a prototype toolkit. Lessons learned include having multiple internal champions at the intervention site, observing workflow pre-intervention, and streamlining data collection with a relational database and visualization software. Insights from the EQUIPPED experience can serve as a model for other systems and collaborative networks.

